# Catwalk: Leopards Persist at Moderate Densities While Exhibiting Temporal Segregation From Humans in Telangana's Boath Range

**DOI:** 10.1002/ece3.73987

**Published:** 2026-07-14

**Authors:** Nilanjan Basu, Imran Siddiqui, Prasanth Bajirao Patil, Maximilian L. Allen

**Affiliations:** ^1^ Hyderabad Tiger Conservation Society (HyTiCoS) Hyderabad Telangana India; ^2^ Centre for Wildlife Studies (CWS) Bengaluru Karnataka India; ^3^ Indian Forest Service, Telangana Forest Department Adilabad Telangana India; ^4^ Illinois Natural History Survey University of Illinois Champaign Illinois USA

**Keywords:** camera trap, coexistence, diel activity, human, population, spatially‐explicit capture‐recapture, Telangana

## Abstract

Large carnivores, such as leopards ( *Panthera pardus* ), are critical for the structure and function of ecosystems but are declining across much of their range. Quantifying their density and behavioural responses in human‐dominated landscapes is therefore important for conservation planning. We estimated the density of leopards and their temporal overlap with prey, humans, livestock and other sympatric predators in human‐dominated forest patches. We deployed 37 camera traps in the forested area of Boath Range of Ichoda Forest Division, located in Adilabad district, Telangana, India. The camera traps were active for ~42 days which yielded 1243 trap nights from December 2024 to January 2025. We captured a total of 51 independent leopard events at 13 locations. Spatially explicit capture‐recapture (SECR) models fit separately to the left and right flanks produced concordant density estimates of 6.37/100 km^2^ (95% CI: 1.89–21.45) and 8.56/100 km^2^ (95% CI: 2.08–35.17), respectively. Analyses of diel activity indicated strong temporal segregation between leopards and humans and livestock, whereas leopards exhibited more temporal overlap with sympatric carnivores and common prey species. Our results suggest that there is a moderate population of leopard persisting within a heavily used landscape, apparently exhibiting temporal partitioning with human risk. However, pervasive human activity could erode this coexistence if left unmanaged. We recommend a holistic approach to human‐leopard coexistence to support conservation and local economies based on sustainable use and potential eco‐tourism in fragmented forest landscapes.

## Graphical abstract

Temporal overlap between male leopards and female leopards in Ajjar‐Wajjar Forest Block, Adilabad District, Telangana, India.

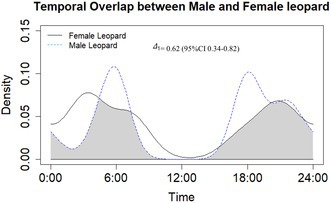

## 1 Introduction

Large carnivores shape ecosystems through top‐down processes that regulate prey populations (Estes et al. 2011) and mediate interactions among competitors (Prugh and Sivy 2020), with cascading effects on biodiversity and ecosystem stability (Ripple et al. 2014; Ford and Goheen 2015). However, these effects vary across landscapes due to differences in prey communities, competitor guilds and human disturbance. Despite their ecological importance and role as umbrella species (Roberge and Angelstam 2004), many large carnivores are declining due to habitat loss, prey depletion and direct persecution (Ripple et al. 2014; Torres‐Romero et al. 2025). Understanding how their densities vary across human‐dominated and intact systems is therefore critical for setting realistic conservation and management targets.

Monitoring large carnivores is inherently challenging because of their low densities, cryptic behaviour and avoidance of humans (Caravaggi et al. 2017). Camera trapping has emerged as a cost‐effective, non‐invasive approach that enables robust ecological inference (Wearn and Glover‐Kapfer 2019). For species with individually identifiable markings, capture–recapture and spatial capture–recapture (SECR) models provide reliable density estimates (Karanth 1995; Karanth and Nichols 1998). Where individual identification is not feasible, approaches such as occupancy modelling and random‐encounter models allow estimation of distribution and relative abundance while accounting for imperfect detection (MacKenzie et al. 2002; Rowcliffe et al. 2008; Burton et al. 2015). Integrating density estimates with temporal activity patterns can further clarify mechanisms of coexistence, particularly in human‐dominated landscapes.

Leopards ( *Panthera pardus* ) are highly adaptable, wide‐ranging felids whose densities vary across their range (Jacobson et al. 2016; Allen et al. 2020), and now occur only in about one‐third of their historic distribution (Jacobson et al. 2016). As generalist predators, they exhibit considerable flexibility in diet, prey selection and hunting strategies (Allen et al. 2026; Hart et al. 1996; Hayward et al. 2006). Leopards may function either as apex predators or as subordinates within carnivore guilds, and they frequently persist outside protected areas in proximity to humans (Allen et al. 2026; Athreya et al. 2016; Ghoddousi et al. 2016). This ecological plasticity likely reflects responses to spatial and temporal variation in prey availability and competition, pressures that are often intensified in human‐modified landscapes (Ghoddousi et al. 2016; Henschel et al. 2011). Consequently, leopards provide an ideal system for examining how bottom‐up (prey) and top‐down (competition and human activity) processes jointly influence carnivore populations.

In India, camera trapping is widely used to monitor large carnivores and their prey to inform conservation and management (Karanth 1995; Kumbhojkar et al. 2020; Tanwar et al. 2021). Long‐term standardised monitoring has provided critical insights into population trends, movement and conservation effectiveness (Karanth et al. 2006; Karanth and Stith 2011). However, most such efforts have focused on protected areas, whereas Reserved Forest divisions outside protected areas, which have lower levels of protection and often function as corridors with high human use, remain understudied, particularly with respect to rigorous density estimation. This represents a key knowledge gap, as such landscapes are critical for connectivity and are sites of frequent human–wildlife interactions.

Our study was conducted in a Reserved Forest landscape in northern Telangana that serves as a corridor linking forested habitats in Maharashtra, including the Tipeshwar Wildlife Sanctuary and Pyanganga Wildlife Sanctuary, with the Kawal Tiger Reserve (Dutta et al. 2018). These multi‐use forests form a mosaic of woodland, agriculture and settlements, where wildlife and humans co‐occur. The presence of leopards, along with occasional tiger movement, underscores the importance of this landscape for both connectivity and interspecific interactions. We hypothesised that smaller forest patches embedded within human‐dominated landscapes function both as resident habitats and dispersal corridors for leopards. We further expected that individuals with higher recapture frequencies would be more likely to have activity centres within or near the sampling area, whereas individuals detected infrequently may represent transient or wide‐ranging individuals. Next, we hypothesised that temporal partitioning with humans and competitors facilitates coexistence. To test this, we pursued two objectives: (1) to estimate leopard density across the study area and (2) to assess temporal interactions between leopards and humans, livestock, prey and sympatric carnivores. Specifically, we predicted that (1) leopard density would be lower than or comparable to values reported from strictly protected forests but sufficient to indicate resident populations and (2) leopards would exhibit crepuscular activity with high overlap with primary ungulate prey, low overlap with humans and livestock and moderate overlap with sympatric carnivores.

## 2 Materials and Methods

### 2.1 Study Area

**FIGURE 1:**
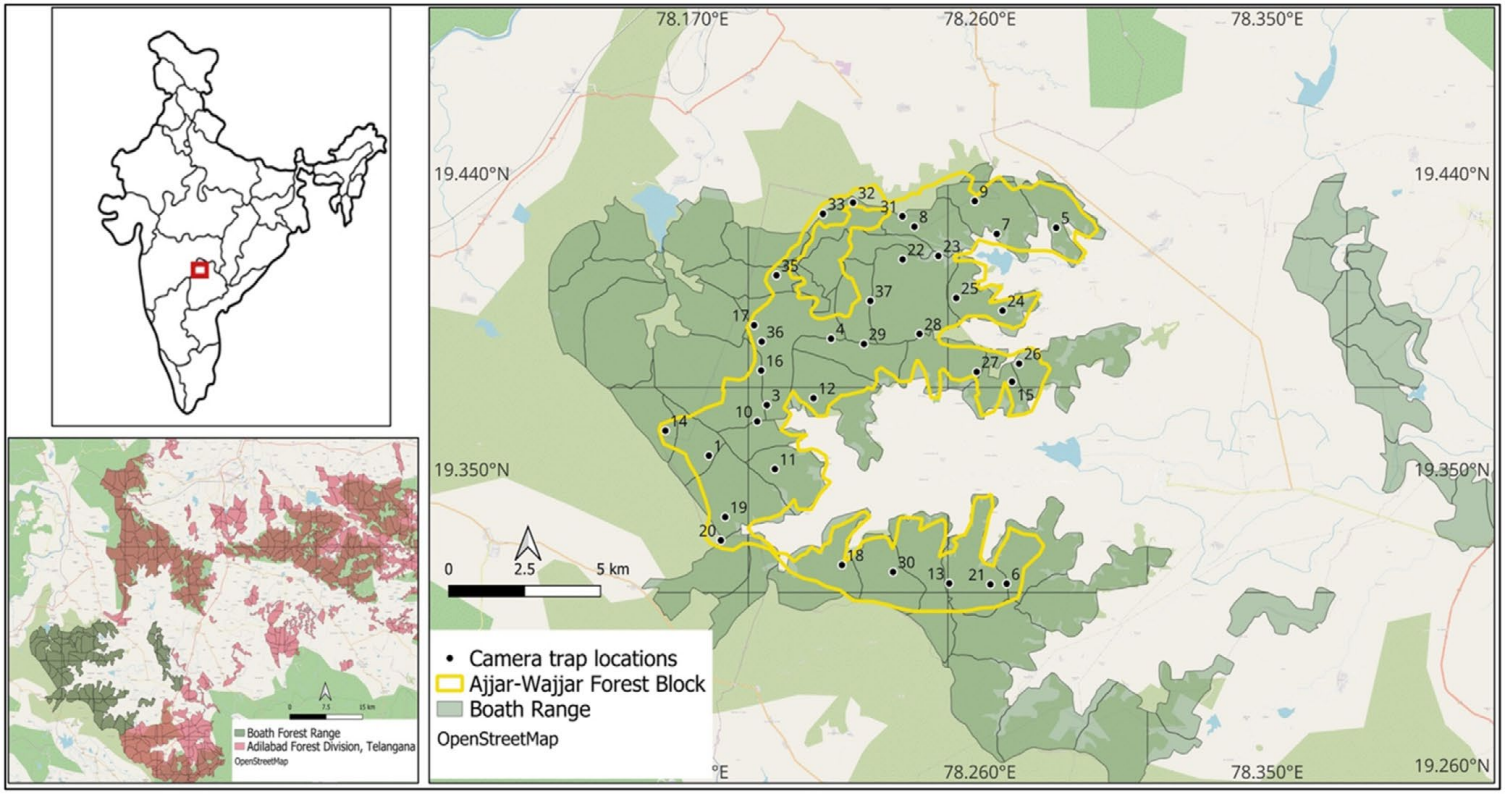
The Ajjar‐Wajjar Forest block in Boath Range, Ichoda Forest Division, Adilabad District, Telangana, India. The dots are the locations of camera traps deployed in the study area.

We conducted our study in the Boath Range, situated in the southern part of Ichoda forest division in Adilabad district of Telangana, near the boundary of the neighbouring state of Maharashtra, in the Central Indian Landscape (Figure 1). The climate was tropical, characterised by temperatures that ranged from 5°C in winter to 43°C in summer and had an annual average rainfall of approximately 1040 mm (Siddiqui 2010). The vegetation was characterised by tropical dry deciduous forest (Champion and Seth 1968), embedded within a village–agriculture mosaic typical of the Central Indian Landscape. We surveyed ~90 km^2^ within the Ajjar–Wajjar forest block of Boath Range, but excluded a small interior hamlet from sampling to avoid privacy and access constraints. The regional large‐mammal assemblage included leopards, grey wolves ( *Canis lupus* ) and, periodically, tigers moving along the Kawal–Tipeshwar interface, with dhole ( *Cuon alpinus* ) recorded in the broader landscape. The presence of omnivores like sloth bear ( *Melursus ursinus* ) had been reported from the area as well. Common ungulates included wild pig ( *Sus scrofa* ), sambar ( *Rusa unicolor* ), nilgai ( *Boselaphus tragocamelus* ) and four‐horned antelope or chousingha ( *Tetracerus quadricornis* ). Chital ( *Axis axis* ) was rare but had been reported earlier. The landscape also supported Scheduled Tribe communities (e.g., Gonds) whose livelihoods were closely linked to forest resources like firewood, medicinal plants, etc. (Mohan et al. 2017).

### 2.2 Field Methods

We deployed 37 unbaited camera traps on wildlife travel routes (i.e., trails, seasonal streams, dirt tracks, forest roads) to maximise the probability of detecting leopards while also sampling their prey and competitors (Sehgal et al. 2022). Before deploying the camera traps, we performed reconnaissance surveys of the areas for signs (i.e., pugmarks, scat, rake marks, scrape marks, pellets) to identify frequently used travel routes. As part of our capacity building for forest staff, we had them involved in collecting data from their respective beats (the smallest administrative compartments), to ensure we maximised capture of the focal species. We followed the protocol prescribed by Karanth and Nichols (1998), by mapping potential locations for the camera traps using QGIS 3.4 and choosing the camera trap locations based on the frequency of animal signs. At each site, we deployed a single camera trap (Cuddeback, Green Bay, Wisconsin, USA) facing the trail, tied onto tree trunks 35–45 cm above the ground. Each camera trap was set to have no delay between triggers, to take 2–3 photographs per trigger and to be operational for 24 h. We gave each camera trap a unique ID and the camera trap recorded time, date and temperature for each photograph. We considered photographic events of the same species independent if separated by ≥ 2 min to avoid pseudo‐replication while ensuring minimal loss of data (Kays and Parsons 2014; Allen et al. 2022). We identified individual leopards from rosette patterns on a single flank following established photo‐ID protocols (Jhala et al. 2020). Because only a single camera was deployed at each station, individuals were not photographed simultaneously on both flanks; therefore, capture histories were constructed separately for left and right flanks, following standard practice in camera trap‐based capture–recapture studies of individually identifiable carnivores (Karanth and Nichols 1998; Efford and Fewster 2013; Jhala et al. 2020). In each case of individual identification, we had two independent reviewers assign individual IDs and we had a third adjudicated any disagreements. We excluded low‐quality images and those we could not identify from capture histories (Karanth 1995).

### 2.3 Density Estimation

We estimated leopard density using spatially‐explicit capture‐recapture (SECR) models using the package ‘SECR’ (Efford and Fewster 2013) in R (version 4.4.3; R Core Team 2025). SECR models animal capture‐recapture data in a framework with individual activity centres as a spatial point process in continuous space (the state model) and models detection as declining with distance from each individual's activity centre (the observation model) (Borchers and Efford 2008). This framework explicitly accounts for variable exposure of camera traps to the home ranges of different individuals and thereby resolves edge effects that bias non‐spatial estimators (Otis et al. 1978; Efford and Fewster 2013). Camera‐traps function as proximity detectors because they can detect multiple animals within an occasion, and they do not detain detected animals, which remain free to be detected by other camera‐traps within each occasion. One critical assumption for closed population estimation is that the population should be demographically and geographically closed (i.e., no emigration or immigration; Otis et al. 1978). We constructed daily capture histories (occasion = 24 h) over a single closed session (~42 days), to satisfy the closure assumptions (Otis et al. 1978). This short‐duration sampling approach is standard in SECR‐based carnivore studies in India, where maintaining closure is prioritised over extended sampling periods that may introduce violation of demographic and geographic closure due to wide‐ranging movements of leopards. We considered multiple captures of the same individual on one occasion at the same camera location as one detection. We compared half‐normal and hazard‐rate detection functions; models incorporating finite mixture heterogeneity (pmix) were evaluated but not retained due to lack of support. We estimated density with the maximum likelihood obtained from the model fitted with ‘SECR’.

### 2.4 Temporal Activity of the Predator and Prey

We pooled detections from all identified leopards (*n* = 30) and classified them by sex (male and female). Temporal activity patterns of leopards and other species were derived from time‐stamped camera trap images and analysed using the ‘overlap’ package (Meredith et al. 2024) in R (version 4.4.3; R Core Team 2025). We followed the framework of Ridout and Linkie (2009) to estimate coefficients of temporal overlap using non‐parametric kernel density estimation. Within the ‘overlap’ package, detection times are treated as a random sample drawn from an underlying distribution that describes the probability of a photograph being recorded at any given time interval throughout the day. The resulting probability density function (PDF) is interpreted as the species' activity pattern, assuming that individuals are equally likely to be photographed at any time when they are active (Ridout and Linkie 2009).

The analysis proceeded in two steps. First, activity patterns were estimated non‐parametrically using kernel density estimation. Bandwidth selection followed the method of Taylor (2008), and 2000 bootstrap samples were used to derive confidence intervals. Second, temporal overlap between pairs of activity distributions was quantified using the coefficient of overlap (Δ; Weitzman 1970), which ranges from 0 (no overlap) to 1 (complete overlap). This coefficient is defined as the integral of the minimum of the two density functions across the 24‐h cycle, representing the shared area under both curves.

These estimators rely on kernel density functions fitted to the data to approximate the underlying distributions f(t) and g(t). Schmid and Schmidt (2006) describe five estimators of overlap: ∆̂₁ is calculated from vectors of densities estimated at T equally spaced time points, t, between 0 and 2π. For circular distributions, ∆̂_2_ is equivalent to ∆̂₁, while ∆̂₃ is not applicable. The estimators ∆̂₄ and ∆̂₅ are derived from vectors of densities evaluated at the observed activity times of the two species, *x* and *y*:

$$\begin{array}{l} {\hat{\Delta }}_{1}=\frac{2\pi }{T}\sum^{T}\min\left\{\hat{f}\left(t_{1}\right)-\hat{g}\left(t_{1}\right)\right\}\\ {\hat{\Delta }}_{4}=\frac{1}{2}\left(\frac{1}{n}\sum_{i=1}^{n}\min\left\{1,\frac{\hat{g}\left(x_{i}\right)}{\hat{f}\left(x_{i}\right)}\right\}+\frac{1}{m}\sum_{j=1}^{m}\min\left\{1,\frac{\hat{f}\left(x_{i}\right)}{\hat{g}\left(x_{j}\right)}\right\}\right)\\ {\hat{\Delta }}_{5}=\frac{1}{n}\sum_{i=1}^{n}1\left\{\hat{f}\left(t_{i}\right)<\hat{g}\left(t_{i}\right)\right\}+\frac{1}{m}\sum_{j=1}^{n}1\left\{\hat{f}\left(y_{j}\right)<\hat{g}\left(y_{j}\right)\right\} \end{array}$$

where n, m are the sample sizes and I is the indicator function (1 if the condition is true, 0 otherwise).

## 3 Results

**FIGURE 2:**
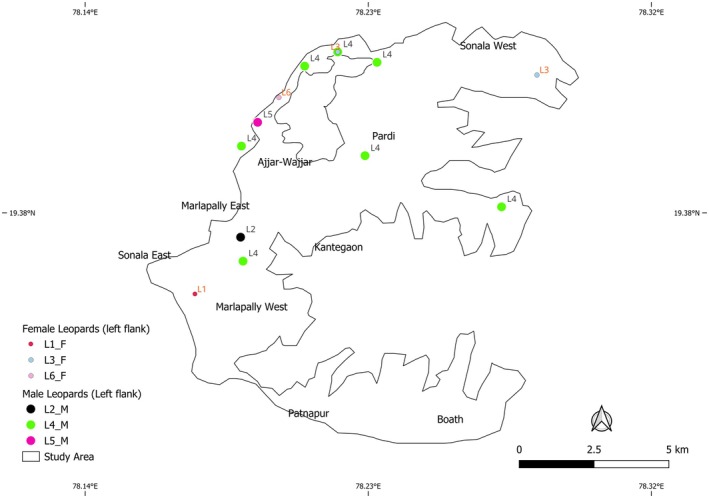
Photo captures of leopards (left flank) in Boath range, Ichoda Division, Adilabad District, Telangana, India. The names written in black are the beats (the smallest administrative Compartment) of the Forest Block. F, Female; M, Male; U, Unidentified sex.

**FIGURE 3:**
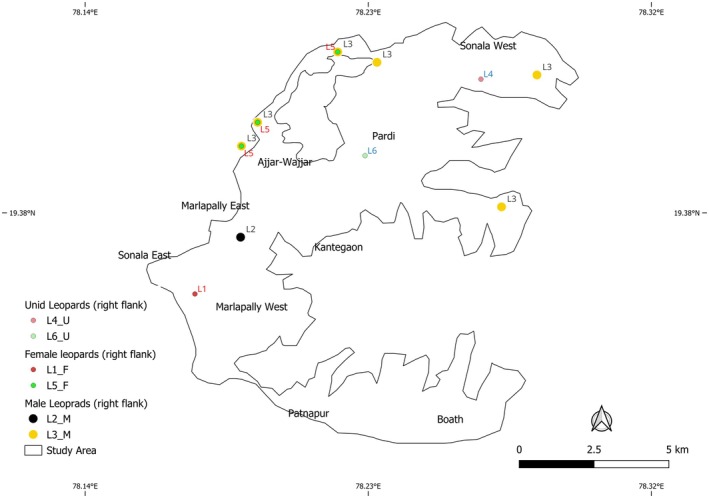
Photo captures of leopards (right flank) in Boath range, Ichoda Division, Adilabad District, Telangana, India. The names written in black are the beats (the smallest administrative Compartment) of the Forest Block. F, Female; M, Male; U, Unidentified sex.

We monitored the 37 camera trap sites for ~42 days yielding a sampling effort of 1243 trap nights. Across that time, we collected a total of 83,074 photos, of which only 2671 were of wild animals. We documented a total of 51 leopard events at 13 sites. The left flank as well as the right flank captures of the leopard recorded 6 unique individuals (1:1 sex ratio; Figures 2 and 3). Capture frequencies varied among individuals, with one male (L4, left flank; *n* = 10) and one female (L5, right flank; *n* = 5) detected more frequently than others, while the remaining individuals were recorded only sporadically.

### 3.1 Leopard Density Estimates

We fit spatially explicit capture–recapture (SECR) models separately to left and right flank data to estimate leopard density (Table 1). For both flanks, the null model with a half‐normal detection function (D ~ 1, g_0_ ~ 1, σ ~ 1) was the most parsimonious, receiving the highest AIC support (Supporting Information). The hazard‐rate model showed nearly identical fit (ΔAIC < 0.01), indicating negligible difference in model performance and suggesting that detection probability declined with distance from activity centres under both formulations.

**TABLE 1:** Final parameter estimates from the selected null models for leopard ( *Panthera pardus* ) density in the study area (*n* = sample size).

Parameters	Left flank (*n* = 16)	Right flank (*n* = 14)
Density (per 100 km^**2**^)	6.37 (95% CI: 1.89–21.45)	8.56 (95% CI: 2.08–35.17)
g_0_ (%)	1.21% (95% CI: 0.47–3.09)	0.71% (95% CI: 0.24–2.07)
σ (km)	6.11 km (95% CI: 3.29–11.32)	5.69 km (95% CI: 2.67–12.13)
Sex ratio	1:1	1:1

Estimated leopard density was 6.37 individuals per 100 km^2^ (95% CI: 1.89–21.45) for the left flank and 8.56 individuals per 100 km^2^ (95% CI: 2.08–35.17) for the right flank. Although point estimates differed between flanks, the confidence intervals overlapped substantially, indicating no meaningful difference between estimates.

### 3.2 Temporal Activity

**FIGURE 4:**
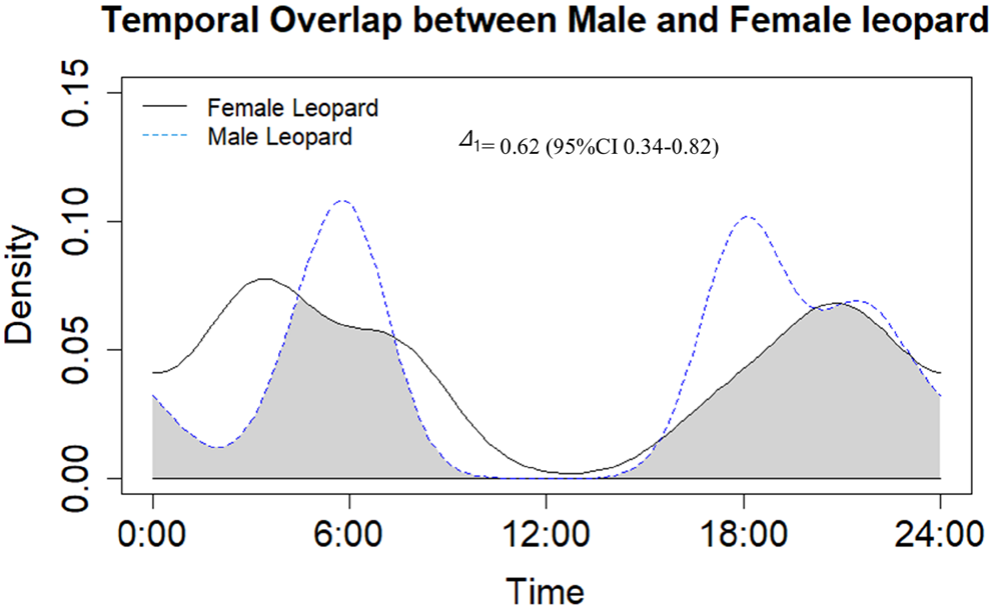
Temporal overlap between male leopard and female leopard in Ajjar‐Wajjar Forest Block, Adilabad District, Telangana, India. The degree of overlap is represented by the $\hat{∆}$_1_ value.

Leopards exhibited a distinct crepuscular activity pattern, characterised by two prominent peaks, one major activity peak around 18:00 h and a secondary peak near 06:00 h, corresponding to the onset of dusk and dawn, respectively. The male leopards showed steep peaks at the above‐mentioned times compared to females (Figure 4). Activity was markedly reduced during midday and early afternoon hours for both males and females, indicating avoidance of high daytime temperatures and human activity periods.

**FIGURE 5:**
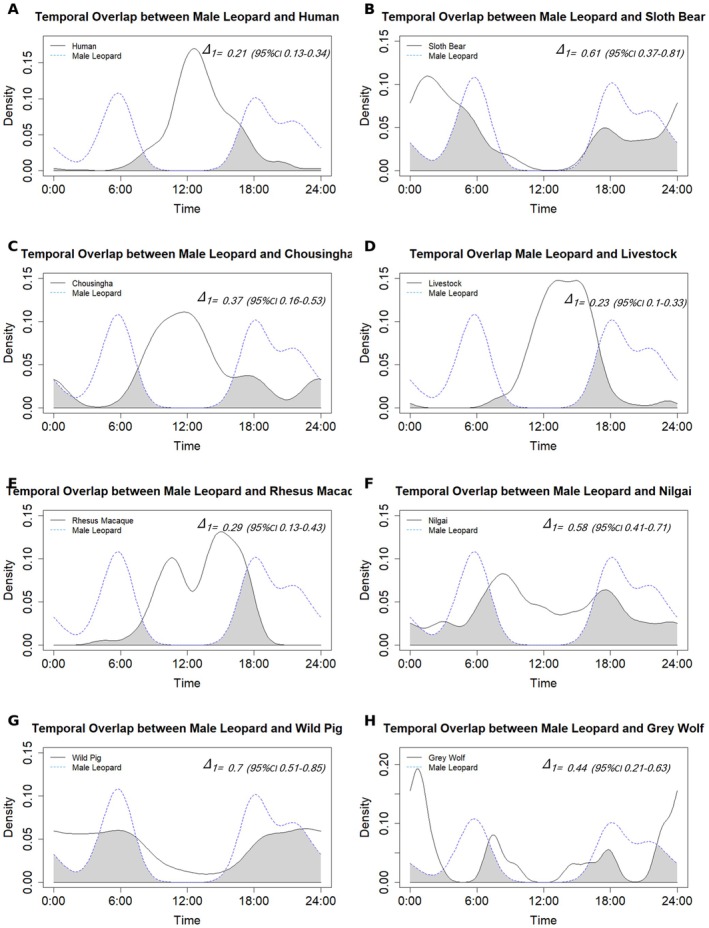
Temporal overlap between male leopard and different species (including humans and livestock) in Ajjar‐Wajjar Forest Block, Adilabad District, Telangana, India. The degree of overlap is represented by the $\hat{∆}$_1_ values.

**FIGURE 6:**
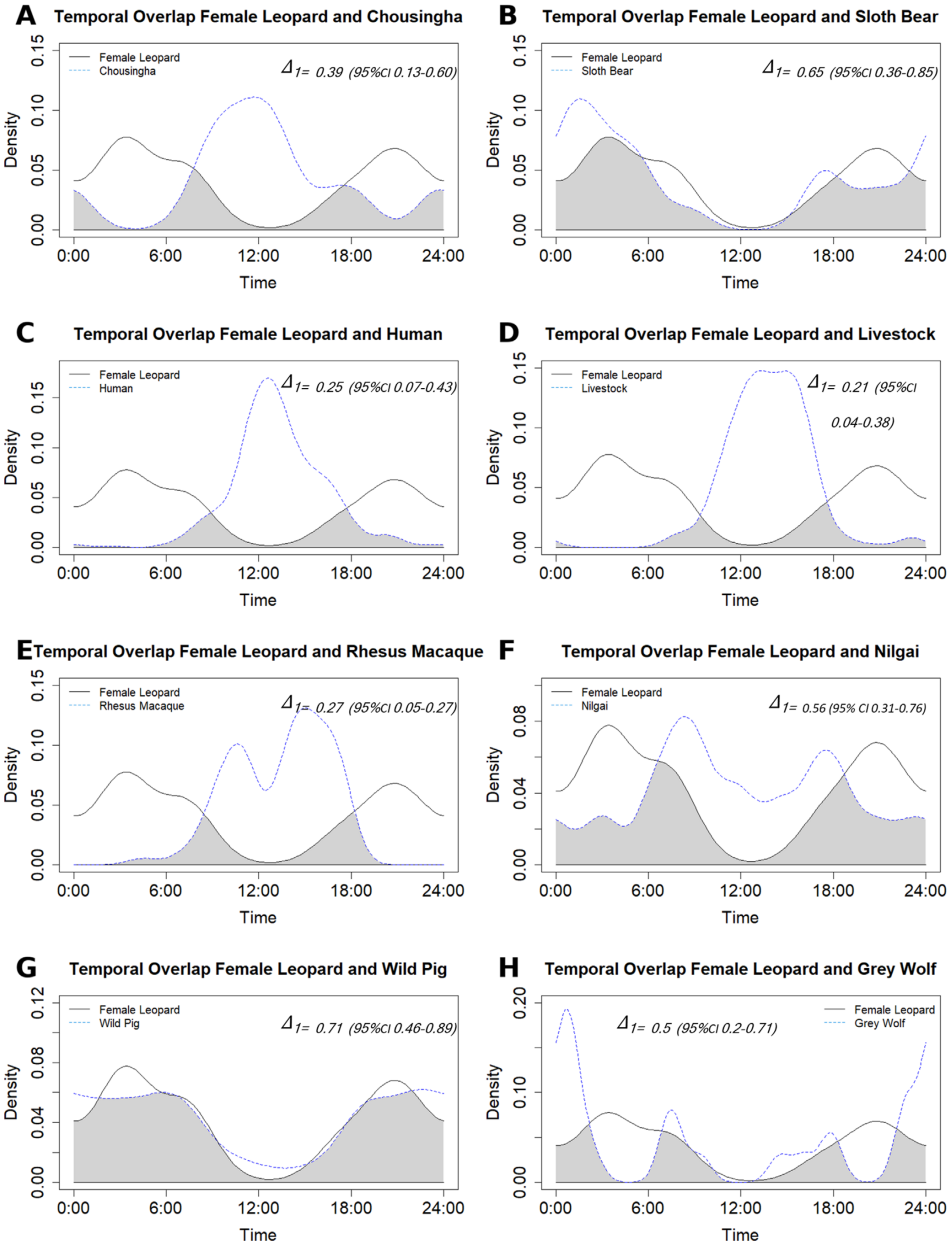
Temporal overlap between female leopard and different species (including humans and livestock) in Ajjar‐Wajjar Forest Block, Adilabad District, Telangana, India. The degree of overlap is represented by the $\hat{∆}$_1_ values.

Our analyses of temporal overlap revealed variable overlap patterns between leopards and their potential prey species. The highest overlap was observed between the female leopards and wild pigs ($\hat{∆}$_1_ = 0.71, 95% CI 0.46–0.89) followed by male leopards and wild pigs ($\hat{∆}$_1_ = 0.70, 95% CI 0.51–0.85). The lowest overlap was observed between the female leopards and rhesus macaques ($\hat{∆}$_1_ = 0.27, 95% CI 0.05–0.48), followed by male leopards and rhesus macaques ($\hat{∆}$_1_ = 0.29, 95% CI 0.13–0.43) (Figures 5 and 6).

Leopards have shown comparatively low overlap with competitor species. Both male and female leopards showed the highest temporal overlap with sloth bears ($\text{Male}\;\hat{∆}$_1_ = 0.61, 95% CI 0.37–0.81; $\text{Female}\hat{∆}$_1_ = 0.65, 95% CI 0.36–0.85). Notably, both males and females had minimal temporal overlap with humans and livestock (Figures 5 and 6).

## 4 Discussion

Camera traps have emerged as an essential wildlife monitoring tool across the world (Burton et al. 2015, 2024), as well as in India, where they provide critical data on animal populations while minimising disturbance to both wildlife and human activities (Karanth et al. 2017). In the Ajjar‐Wajjar Forest Block, Boath Range, our camera trap study revealed important insights about leopard populations and their coexistence with other species and human communities. Specifically, there appears to be a moderate population of leopard persisting within a heavily used human activity matrix, showing temporal segregation from the human activity. Unlike protected areas where these tools are routinely used for monitoring charismatic species like tigers (Jhala et al. 2020), our findings in this human‐dominated landscape highlight both conservation challenges and opportunities that require thoughtful management interventions. These results offer a baseline against which managers can evaluate the population trend, potential risk and the effectiveness of management actions over time in the area.

Population estimation is essential for management practices and recent studies conducted on leopard population estimation in the mosaic, fragmented forest landscapes of Central India have recorded a population of 23.0 (SE 4.8) leopards in Jabalpur and 17.6 (SE 5.4) leopards in Indore, Madhya Pradesh (Majumder et al. 2024). The reported estimated leopard density in Sanjay Gandhi National Park and Tungareshwar Wildlife Sanctuary, Maharashtra, is 26.34 (SE 4.96)/100 km^2^ and 5.40 (SE 2.99)/100 km^2^, respectively (Surve et al. 2022). The protected area with similar anthropogenic stressors, Asola Bhatti Wildlife Sanctuary (size 32 km^2^), in Delhi has reported the leopard density of 4.5 (SE 0.01)/100 km^2^ (Chaudhary et al. 2023). Therefore, our estimated density of approximately 6–9 leopards per 100 km^2^ in the study area can qualify as moderate. These estimates were consistent across flank‐specific analyses, with overlapping confidence intervals indicating no meaningful difference between model outputs. This is a promising find for the persistence of leopards in the Ajjar‐Wajjar forest block, especially when the leopard population in Telangana State declined from 334 (SE 16) in 2018 to 297 (SE 20) in 2022 (Qureshi et al. 2024).

Models incorporating detection heterogeneity (finite mixture models) were evaluated but received substantially less support (ΔAIC ≈ 4) compared to the null model (Supporting Information). As these models did not improve model fit or inference, we based our interpretations on the more parsimonious null model. The Ajjar–Wajjar forest block is connected to a larger forested landscape in the Kinwat Forest Division and the Pyanganga Wildlife Sanctuary, which may facilitate movement of individual leopards across the region. The relatively low detection probability (g_0_) and large spatial scale parameter (σ > 5 km) likely reflect both leopard movement ecology and the limited spatial recapture structure of the dataset. Accordingly, these parameter estimates should be interpreted cautiously and not solely as direct indicators of movement behaviour.

Notably, capture frequencies varied among individuals, with one male and one female being detected more frequently than the others. This pattern may suggest that these individuals had activity centres closer to the sampling area and therefore exhibited greater site fidelity. However, given the limited sample size, this inference remains tentative.

As generalist predators, leopards are often able to persist in human‐dominated landscapes (Athreya et al. 2013; Odden et al. 2014). We hypothesised that the interactions of leopards with humans, prey and competitors in a shared landscape would facilitate coexistence through behavioural adjustments of diel activity. In heterogeneous or mosaic landscapes, leopards often exploit domestic animals as prey when the availability of wild prey is low (Kshettry et al. 2018; Majumder et al. 2025), yet they tend to avoid areas with high human presence, indicating a behavioural trade‐off between prey acquisition and risk avoidance (Palei et al. 2022). Our observations corroborate this pattern, showing that both male and female leopards in the study area actively avoid humans and livestock, resulting in minimal temporal overlap with these groups (Figures 5 and 6). Conversely, they exhibited the highest temporal overlap with abundant wild ungulates, particularly wild pigs, highlighting a potential preference for accessible natural prey. Male leopards showed greater temporal overlap with larger prey species, such as nilgai, than females, which may indicate a female preference for smaller prey species (Voigt et al. 2018). The male and female leopards showed 62% temporal overlap; however, females avoided overlap with males during steep peaks (Figure 4). This could indicate that females may avoid competition from males when they are highly active (Havmøller et al. 2020). Additionally, both sexes showed greater temporal overlap with potential competitors such as sloth bears and grey wolves (Figures 5 and 6) than with humans or livestock. Females showed greater overlap with competitors than males, indicating that males avoided competitors in the study area. This suggests that their spatial and temporal activity patterns are shaped by a complex interplay of prey selection, interspecific competition and human avoidance (Odden et al. 2014; Athreya et al. 2016; Jacobson et al. 2016). This behavioural flexibility likely promotes leopard persistence and coexistence within human‐dominated landscapes, highlighting the need for the protection of several small forest patches in human‐dominated landscapes, along with single large protected areas.

Our inference is constrained by a relatively small sample size and limited number of uniquely identified individuals. While spatial capture–recapture and kernel density methods are generally robust to moderate sample sizes, behavioural interpretations (e.g., sex differences in prey use and temporal segregation) should be considered exploratory. Longer‐term monitoring would be required to determine whether these patterns are stable over time or reflect short‐term spatiotemporal variation.

### 4.1 Conservation Implications

Human activity in the forest presents both challenges and opportunities for conservation (Braczkowski et al. 2018). Our cameras detected frequent movement of people, livestock and domestic dogs throughout the study area, indicating high levels of human use and the need for conservation strategies that actively involve local communities. The low number of detections of wild ungulates (i.e., nilgai, chousingha), along with the near absence of chital, an important component of leopard diets in Central Indian dry‐deciduous ecosystems (Mondal et al. 2012), suggests limited availability of key prey species. Compared to other forested landscapes in Central India, where ungulate communities are typically dominated by chital and sambar, this pattern indicates relatively lower prey availability in the study area. However, the ungulate detectability may have been influenced by carnivore‐focused camera trap placements. Future work incorporating advanced methodologies like camera trap‐based distance sampling (Howe et al. 2017) would provide more robust estimates of prey density and help clarify its role in shaping predator dynamics in this landscape.

This prey deficiency likely limits how many leopards the forest can support and may be contributing to occasional conflicts with livestock. Any conservation initiative in such areas must therefore include the social carrying capacity along with the ecological carrying capacity (Carter and Linnell 2023). The forest's connectivity between Telangana and Maharashtra makes it ecologically valuable as a wildlife corridor (Wikramanayake et al. 2011); therefore, this study can be used as baseline to formulate conservation efforts by the managers that should be comprehensively planned to ensure long‐term ecological and management outcomes.

Our results indicate that leopards persist within a human‐dominated forest mosaic, with activity patterns that result in limited temporal overlap with humans. While this pattern is consistent with the species' broadly crepuscular behaviour, it may nonetheless reduce the likelihood of direct encounters in multi‐use landscapes. These findings suggest that maintaining forested patches within such landscapes may be important for supporting leopard presence and facilitating spatial and temporal separation from human activity. Approaches that minimise human‐animal conflict by reducing the probability of direct encounters, particularly during peak human activity periods and involving local communities in conservation action have been shown to contribute to coexistence in similar systems (Karanth et al. 2018). Although our study did not directly assess conflict, the observed patterns underscore the importance of continued monitoring of both wildlife and human activity to better understand interaction dynamics. Overall, our findings provide a baseline for understanding leopard occurrence and activity patterns in a multi‐use forest system and longer‐term monitoring will be important to assess the stability of these patterns and inform management strategies in landscapes where wildlife and human activities overlap.

## Author Contributions

**Nilanjan Basu:** data curation (lead), formal analysis (lead), methodology (lead), software (lead), writing – original draft (lead), writing – review and editing (equal). **Imran Siddiqui:** data curation (equal), formal analysis (equal), funding acquisition (lead), investigation (equal), methodology (equal), validation (equal). **Prasanth Bajirao Patil:** funding acquisition (equal), project administration (lead). **Maximilian L. Allen:** conceptualization (lead), supervision (lead), validation (lead), writing – original draft (supporting), writing – review and editing (equal).

## Funding

This work was supported by Gland Fosun Foundation.

## Conflicts of Interest

The authors declare no conflicts of interest.

## Supporting information


**Data S1:** Supporting Information.

## Data Availability

The data used in this study are available as Supporting Information.
